# 190. Performance of urinalysis parameters in predicting significant bacteriuria: Making the case for a population-specific approach to diagnostic stewardship

**DOI:** 10.1093/ofid/ofac492.268

**Published:** 2022-12-15

**Authors:** Sonali D Advani, Nicholas A Turner, Kenneth E Schmader, Rebekah Wrenn, Rebekah W Moehring, Christopher R Polage, Valerie Vaughn, Deverick J Anderson

**Affiliations:** Duke University School of Medicine, Durham, North Carolina; Duke University Medical Center, Durham, North Carolina; Duke and Durham VA Medical Centers, Durham, North Carolina; Duke University, Durham, North Carolina; Duke University, Durham, North Carolina; Duke University School of Medicine, Durham, North Carolina; University of Utah Medical School, Salt Lake City, Utah; Duke University, Durham, North Carolina

## Abstract

**Background:**

Clinicians and laboratories routinely use urinalysis (UA) results to help determine if urine cultures and/or antimicrobials are indicated. Yet, the performance of individual UA parameters and common clinical thresholds for action are not well defined and may vary across different patient populations. Our objective was to compare the performance of different UA parameters in predicting significant bacteriuria irrespective of symptoms, and to assess performance of pyuria based on age, sex, and presence of indwelling catheter.

**Methods:**

This retrospective review of UA and urine culture data from the Duke University Health System included all UAs ordered within 24 hours of a urine culture between 2015 and 2020 (no reflex urine cultures included). We defined significant bacteriuria as a urine culture with ≥1 uropathogen growing at ≥100,000 colony forming units/mL. Then, we used this definition to evaluate the performance of relevant UA parameters and result thresholds including sensitivity, specificity, negative predictive value (NPV) and positive predictive value (PPV). We also combined 18 different UA criteria (as shown in Figure) and used receiver operating characteristic (ROC) curves to identify the top 5 performing models for predicting significant bacteriuria (sensitivity and specificity).

18 Different Combinations of UA Parameters for Predicting Significant Bacteriuria on Urine Cultures

**Figure:**
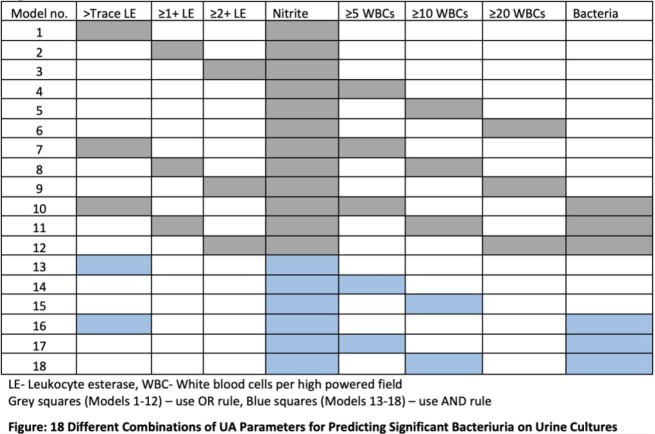

**Results:**

Of 240,195 encounters during the 6-year study period, 38% were outpatient and 62% were inpatient. Twenty-nine percent had a urine culture with significant bacteriuria; 30.7% had a negative urine culture. No single UA parameter had both - high sensitivity and high specificity in predicting bacteriuria. Trace leukocyte esterase and low-level pyuria had a high NPV for significant bacteriuria (Table 1A). Combined UA parameters did not perform better than pyuria alone (Table 1B). The high NPV >=0.90 of pyuria was maintained among most patient age and sex subgroups with the exception of females ≥65 and patients with indwelling catheters (Table 2).

**Table 1A:**
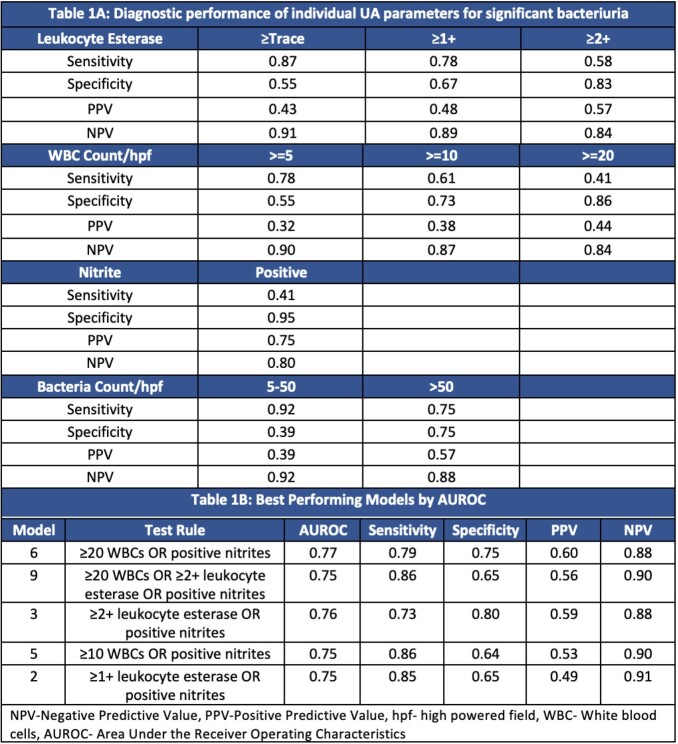
Performance of UA parameters in predicting significant bacteriuria, Table 1B: Best performing models by AUROC after testing 18 models

**Figure:**
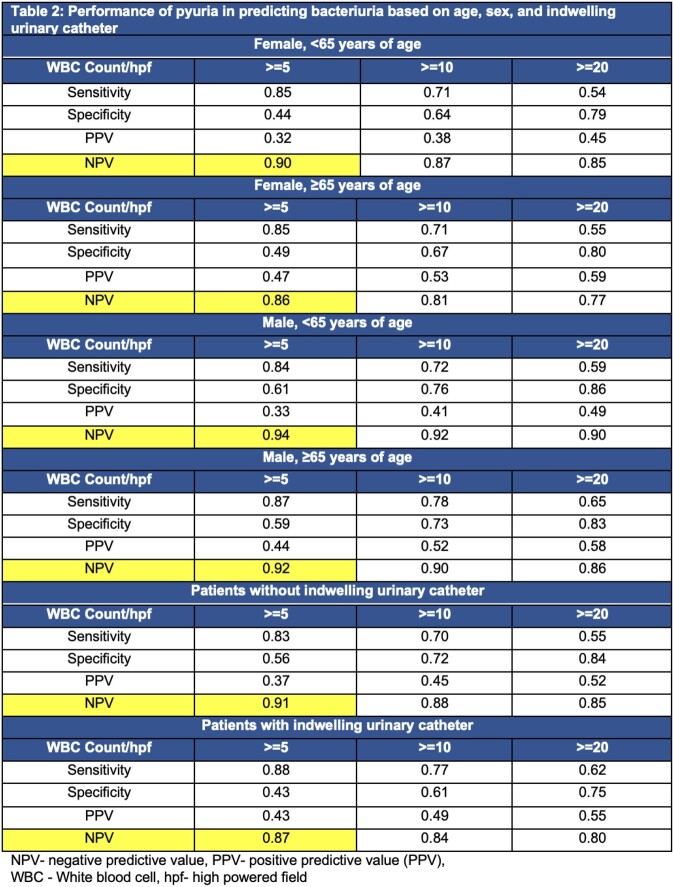

**Conclusion:**

UA parameters should be leveraged for their NPV instead of sensitivity, when used as a part of diagnostic workup. Future reflex urine culture workflows and diagnostic stewardship algorithms should incorporate population-specific UA criteria and/or focus on populations where NPV of pyuria is high.

**Disclosures:**

**Sonali D. Advani, MBBS, MPH, FIDSA**, Locus Biosciences: Advisor/Consultant|Locus Biosciences: Honoraria|Sysmex America: Advisor/Consultant **Nicholas A. Turner, MD, MHSc**, Aperio: Advisor/Consultant **Rebekah W. Moehring, MD, MPH, FIDSA, FSHEA**, UpToDate, Inc.: Author Royalties **Valerie Vaughn, MD, MSc**, Thermo Fisher Scientific: Honoraria.

